# Tuberculosis

**Published:** 1895-10

**Authors:** 


### CONTROL WORK.

### TUBERCULOSIS.

*Massachusetts.*—The tuberculosis question threatens to become a political issue in some sections. Much work in the line of investigation is being done around Marlboro, and all cattle responding to the tuberculin-test were found tubercular on examination. Those destroyed at Leominster on post-mortem were found seriously and extensively affected, remaining condemned cattle ordered to yards at Watertown for destruction. At Cheshire one examined, diseased, and destroyed. At Shelburne, 17 out of 21 milch cows; 10 out of 20 young cattle condemned and destroyed.

*Vermont.*—From March 8th to August 15th, 615 cattle tested; 6 per cent., or 37 animals, reacted. All were killed, one-half value paid, making an average of $16 per head. At Barre animals were tested and condemned.

*New York.*—At Islip, Long Island, 2 destroyed, both Jerseys.

*Pennsylvania.*—At Greenville tuberculin-test applied, results satisfactory.

*Illinois.*—The Chicago *Record* calls for a thorough examination of the herds affording Chicago her milk-supply. One Jersey cow at Levant found tubercular and destroyed.

*Minnesota.*—Dr. M. H. Reynolds, in his plan of education, tested two animals at the State Fair Grounds, and post-mortem held so that the subject might be brought more forcibly to the attention of live-stock owners and dairymen.

